# Increased Nrf2 expression by renal cell carcinoma is associated with postoperative chronic kidney disease and an unfavorable prognosis

**DOI:** 10.18632/oncotarget.25322

**Published:** 2018-06-19

**Authors:** Hideo Yuki, Takao Kamai, Satoshi Murakami, Satoru Higashi, Takahiro Narimatsu, Tsunehito Kambara, Hironori Betsunoh, Hideyuki Abe, Kyoko Arai, Hiromichi Shirataki, Ken-Ichiro Yoshida

**Affiliations:** ^1^ Department of Urology, Dokkyo Medical University, Mibu, Tochigi, Japan; ^2^ Division of Field Application, Life Technologies, Tokyo, Japan; ^3^ Department of Molecular and Cell Biology, Dokkyo Medical University, Mibu, Tochigi, Japan

**Keywords:** nuclear factor erythroid 2–related factor 2 (Nrf2), chronic kidney disease (CKD), renal cell cancer

## Abstract

Chronic kidney disease (CKD) is a worldwide health problem, and prevention of CKD is important for preservation of renal function after kidney surgery. There is evidence that transcription factor nuclear factor erythroid 2–related factor 2 (Nrf2) has a vital antioxidant and detoxifying role in protecting the kidneys against various diseases. Impaired activation of Nrf2 is associated with oxidative stress related to CKD, and Nrf2 is also a key player in the development of cancer. However, the clinical impact of Nrf2 has not been investigated in patients with renal cell carcinoma (RCC). A retrospective study was performed in 89 patients undergoing nephrectomy for RCC. The estimated glomerular filtration rate (eGFR) and serum uric acid (SUA) were investigated over time after surgery. We investigated Nrf2 protein expression in all tumors and single nucleotide polymorphisms (SNPs) of the Nrf2 gene in 7 tumors. In patients whose tumors showed higher Nrf2 expression, there was a more rapid decrease of eGFR and increase of SUA after nephrectomy. Multivariate analysis confirmed that increased Nrf2 expression was an independent poor prognostic factor related to shorter overall survival. Among the 7 tumor samples, an SNP on exon 5 of the Nrf2 gene in one tumor and three genotypes (C/C, C/A, and A/A) of rs6721961 at the promoter region of the Nrf2 gene were observed. Although the mechanisms underlying the influence of Nrf2 are still unclear, our findings suggested that elevated tumor expression of Nrf2 was associated with postoperative CKD and biologically aggressive RCC with an unfavorable prognosis.

## INTRODUCTION

Chronic kidney disease (CKD) is an important health problem worldwide [1]. The glomerular filtration rate (GFR) is regarded as the best overall measure of renal function. Current clinical practice guidelines define chronic kidney disease as an estimated glomerular filtration rate (eGFR) < 60 mL/min/1·73 m^2^, or by detection of markers of kidney damage (such as albuminuria or abnormal imaging studies) for at least 3 months [2]. Despite various advances in slowing the progression of CKD, the incidence of end-stage renal disease (ESRD) is still increasing around the world, with a severe impact on both patients and society [1]. Renal cell carcinoma (RCC) accounts for 3-4% of all malignancies and its incidence is also rising steadily worldwide. Surgical resection of the affected kidney (radical or partial nephrectomy) is the mainstay for achieving local control of RCC [3]. However, it is well known that kidney function is affected by renal surgery [4, 5], and we have encountered RCC patients showing a decrease of eGFR and increase of serum uric acid (SUA) after nephrectomy who eventually developed CKD. Therefore, preservation of renal function after nephrectomy for prevention of CKD is an important issue, and better understanding of the mechanisms underlying postoperative occurrence of CKD in RCC patients is required.

Oxidative stress and inflammation are involved in the development and progression of CKD and its complications, with these two processes being inseparably linked as each provokes and amplifies the other [6]. Uric acid (UA) is a marker of oxidative stress since the pathway for UA production includes xanthine oxidase, an enzyme also involved in producing reactive oxygen species (ROS). In the presence of oxidative stress, UA may contribute to impairment of vascular endothelial function by inhibition of nitric oxide. UA also acts as an antioxidant, free radical scavenger, and chelator of transitional metal ions, which are converted to weakly reactive forms. Oxidative stress associated with CKD is the result of both increased ROS production and diminished antioxidant capacity [7]. Impaired activation of nuclear factor erythroid 2–related factor 2 (Nrf2), the transcription factor that regulates genes encoding antioxidant and detoxifying molecules, is one of the important molecular mechanisms underlying oxidative stress [7]. Cells are subjected to numerous endogenous and exogenous stresses, and there is increasing evidence that Nrf2 has a cytoprotective effect. The Kelch-like ECH-associated protein 1 (Keap1) - Nrf2 pathway is the major regulator of cytoprotective responses to oxidative and electrophilic stress. Under physiological conditions, Keap1 binds to Nrf2, triggering its proteasomal degradation. In the presence of oxidative stress, Nrf2 escapes such degradation and undergoes translocation to the nucleus, where it binds to antioxidant responsive elements (AREs) and up-regulates the expression of various downstream genes [8]. Activation of the Nrf2/ARE axis induces a strong antioxidant response, making pharmacological activation of this pathway a promising target for various diseases, including kidney disease. There is considerable evidence supporting a vital physiological role of Nrf2 in protecting the kidneys, and pharmacological induction of Nrf2 by bardoxolone methyl (methyl-2-cyano 3, 12-dioxooleano-1, 9-dien-28-oate, CDDO-Me), which activates Nrf2 via inhibition of Keap1, seems to be promising for various renal pathologies [9, 10]. Thus, the Nrf2 pathway is thought to be important for protection against redox-mediated injury.

Cancer cells are exposed to high levels of ROS and protection against oxidative stress is essential for their survival, with constitutive activation of Nrf2 being an important mechanism for tumors to adapt to a pro-oxidant environment. As well as regulating oxidative stress, Nrf2 is involved in many other processes, including DNA repair, anabolism, angiogenesis, and acquisition of chemoresistance by cancer cells. In addition, it was recently reported that Nrf2 activates the pentose phosphate pathway and diverts glucose metabolites toward de novo nucleotide synthesis in proliferating cells [11], suggesting that constitutive activation of Nrf2 could be important in the development and progression of human cancers. It seems that Nrf2 has a dual role and promotes either prevention or progression cancer depending on the cellular context and microenvironment [12–14]. These reports suggested that Nrf2 may have a role in the progression of RCC and postoperative CKD. Accordingly, we investigated the association between tumor tissue expression of Nrf2 and various clinicopathological features or renal function in RCC patients undergoing nephrectomy. To our knowledge, this was the first investigation of the role of Nrf2 in postoperative CKD and progression of RCC.

## RESULTS

### Postoperative changes of eGFR and SUA

**Figure 1:**
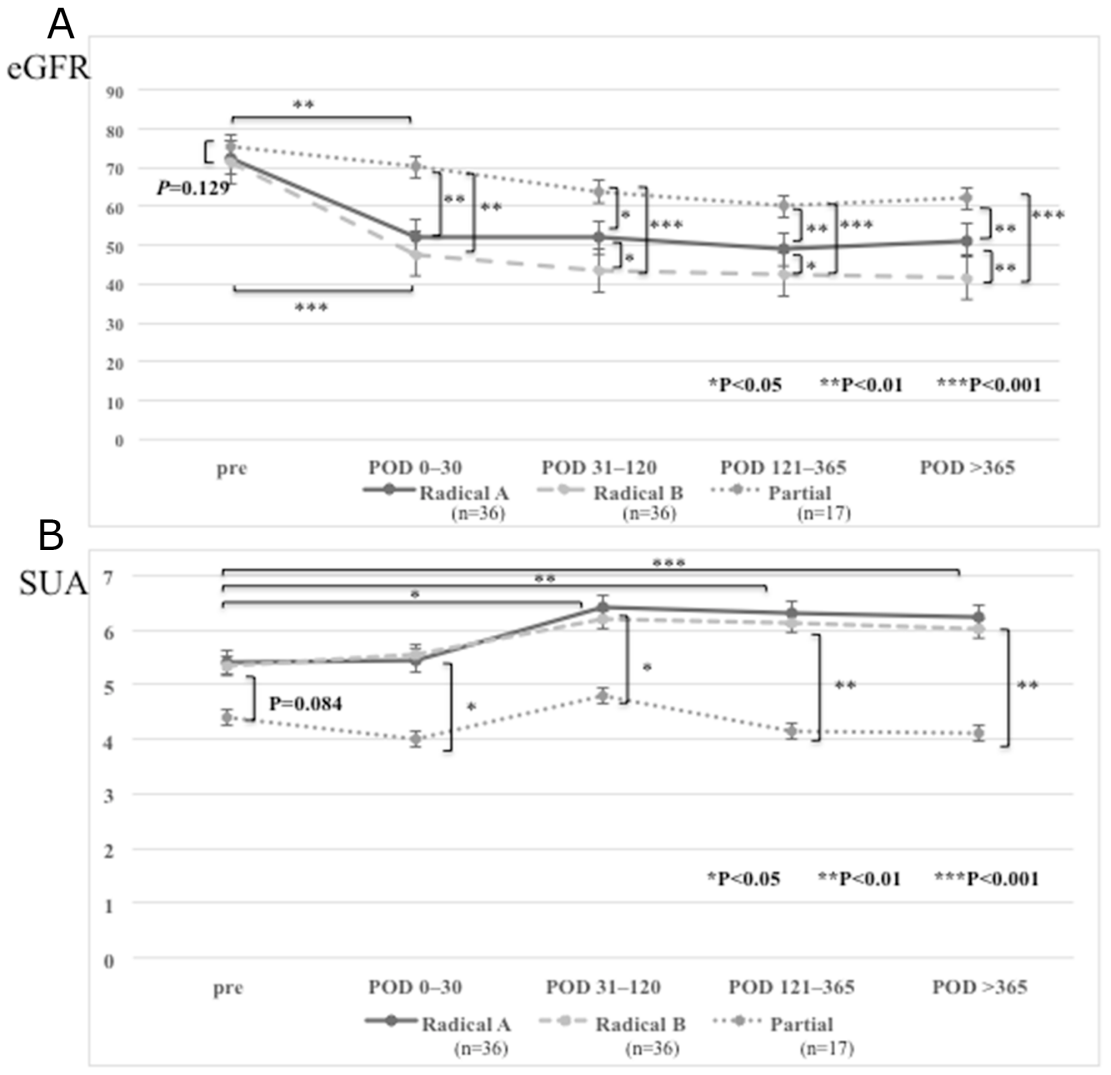
Estimated glomerular filtration rate (eGFR) and serum uric acid (SUA) levels in the patients who underwent a partial or radical nephrectomies **(A)** eGFR was significantly higher in the patients who underwent partial nephrectomy than those who underwent radical nephrectomy in postoperative periods: postoperative days (POD) 0–30, POD 31–120, POD 121–365, and POD >365. In both radical or partial nephrectomy group, postoperative eGFR value decreased compared to preoperative eGFR. The patients who underwent radical nephrectomy with higher Nrf2 expression in the primary tumor (radical B group) showed lower postoperative eGFR than those with lower Nrf2 expression (radical A group). **(B)** SUA levels were significantly lower in the patients who underwent partial nephrectomy than those who underwent radical nephrectomy in postoperative periods. SUA levels in the patients with radical nephrectomy increased after surgery and remained stable at higher levels, while those with partial nephrectomy temporarily increased after surgery but gradually decreased. There was no difference of postoperative SUA levels between in radical A and in radical B groups.

Preoperative eGFR was slightly higher in the patients undergoing partial nephrectomy (partial nephrectomy group; n=17) than in those undergoing radical nephrectomy (radical nephrectomy group; n=72), but the difference was not significant (P = 0.129, Figure 1A). While eGFR decreased in both groups after surgery, it decreased more rapidly in the radical nephrectomy group compared to the partial nephrectomy group. When postoperative and preoperative values were compared, the radical nephrectomy group showed a larger percent decrease of eGFR after surgery than the partial nephrectomy group.

The radical nephrectomy group was divided into two subgroups by the median level of Nrf2 expression in the primary tumor shown by western blotting (median: 3.3), a radical A subgroup with lower expression and a radical B subgroup with higher expression. Postoperative eGFR was lower in the radical B subgroup than in the radical A subgroup (Figure 1A). However, no difference of postoperative eGFR was observed when we similarly divided the partial nephrectomy group into two subgroups by the median level of Nrf2 expression (median: 1.4), probably because of lower median level of Nrf2 expression and smaller number of patients in this group. Therefore, we analyzed the partial nephrectomy group without dividing it.

The preoperative SUA level was higher in the radical nephrectomy group than the partial nephrectomy group, but there was no significant difference (P = 0.084) (Figure 1B). SUA increased postoperatively in the radical nephrectomy group, but not in the partial nephrectomy group, and SUA was significantly higher after surgery in the radical nephrectomy group compared with the partial nephrectomy group.

In the radical nephrectomy group, the postoperative SUA level showed no difference between the radical A subgroup with lower Nrf2 expression in the primary tumor and the radical B subgroup with higher Nrf2 expression (Figure 1B). In the partial nephrectomy group, there was no difference between the two subgroups based on median Nrf2 expression as was the case eGFR, so we again analyzed the partial nephrectomy group without dividing it.

### Association between changes of eGFR or SUA and clinicopathologic characteristics

When the percent change of eGFR from the preoperative level to 1 year after nephrectomy was calculated, the postoperative percent decrease of eGFR was larger (>20%) in the patients with a higher pT stage, less differentiated tumors, and metastasis, but not in those with vascular invasion (Table 1). With regard to SUA, a larger postoperative percent increase (> 10%) was associated with metastasis, but was not associated with histological differentiation, pT stage, or vascular invasion (Table 1).

**Table 1:** Association of changing ratio of eGFR and serum UA with pathologic characteristics

		|ΔeGFR|^*^ < |-20%|	|ΔeGFR|^*^ > |-20%|		|ΔUA|^*^ < 10%	|ΔUA|^*^ > 10%	
Grade	Grade 1	7	2	*P* = 0.0483	7	2	*P* = 0.1492
	Grade 2	11	18		16	13	
	Grade 3	13	38		19	32	
pT	pT1/2	14	8	*P* = 0.0412	14	8	*P* = 0.1353
	pT3/4	17	50		28	39	
Vascular invasion	v0	4	7	*P* = 0.9707	7	4	*P* = 0.4092
	v1	27	51		35	43	
Metastasis	M0	19	6	*P* < 0.0001	22	3	*P* < 0.0001
	M1	12	52		19	45	
$$\text{Decreased ratio of eGFR}{\left(\text{eGFR}\right)}^{*}=\frac{\text{preoperative}-\text{postoperative serum level}}{\text{preoperative serum level}}\text{×}100\left(\%\right)$$							
$$\text{Increased ratio of serum UA}{\left(\text{UA}\right)}^{*}=\frac{\text{preoperative}-\text{postoperative serum level}}{\text{preoperative serum level}}\text{×}100\left(\%\right)$$							

**Figure 2:**
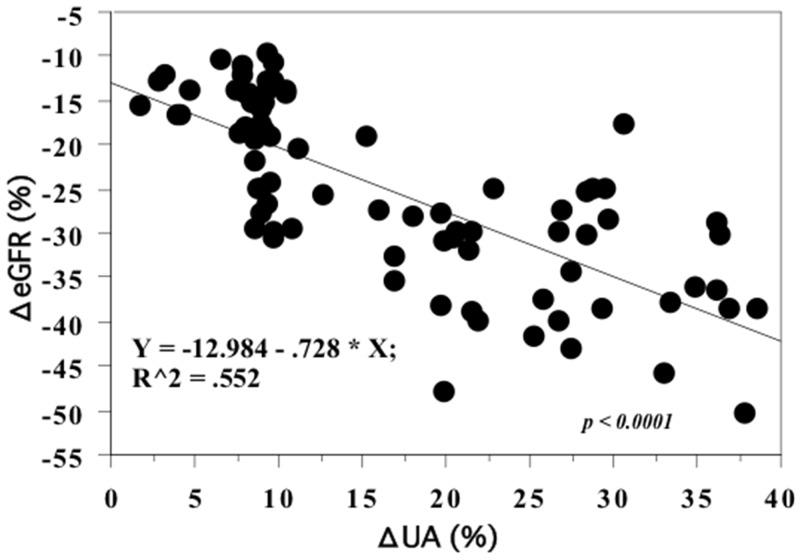
Spearman rank correlation between the percent change of eGFR and SUA between preoperative and 1-year postoperative values The larger increased percent change of SUA showed the significant decreased percent change of eGFR.

In addition, a larger postoperative percent increase of SUA showed a significant positive correlation with a larger postoperative percent decrease of eGFR (r^2^ = 0.55, P < 0.0001, Figure 2).

**Figure 3:**
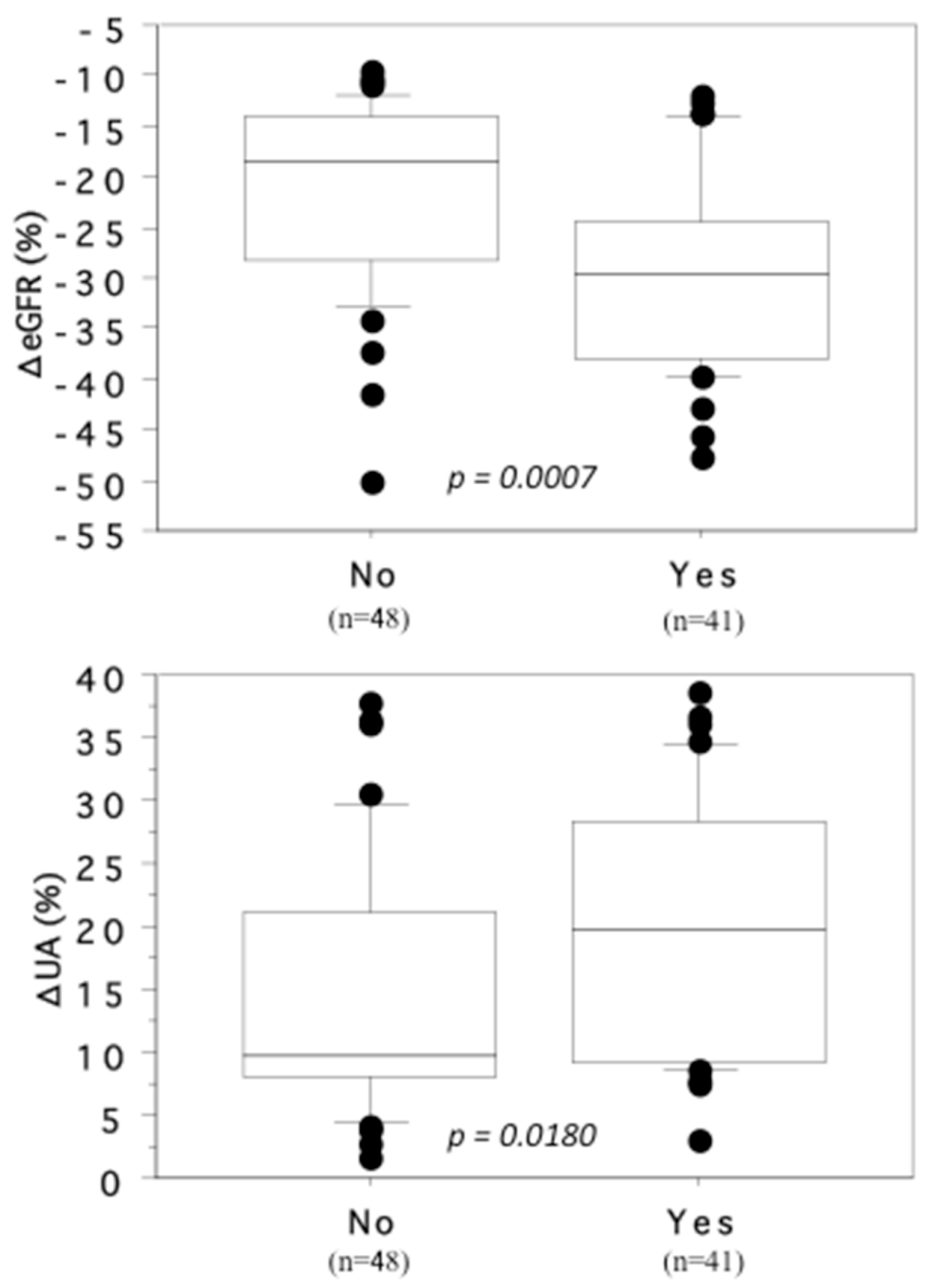
Association of the changing rates of ΔeGFR and ΔSUA with cardiovascular disease, hypertension or diabetes mellitus The patients who received medical treatment for cardiovascular disease, hypertension or diabetes mellitus (Yes) showed the larger decrease of ΔeGFR and the larger increase of ΔSUA than those did not (No).

When the influence of comorbidities (including cardiovascular disease, hypertension, and diabetes mellitus) was assessed, the percent decrease of eGFR and percent increase of SUA were both larger in patients with than without such comorbidities (ΔeGFR: mean ± SD = -29.6 ± 9.51 and -21.7 ± 9.3, respectively, P = 0.0007; ΔSUA: 19.7 ± 10.3 and 14.7 ± 10.0, respectively, P = 0.0180, Figure 3).

### Association of Nrf2 expression with clinicopathologic characteristics

**Figure 4:**
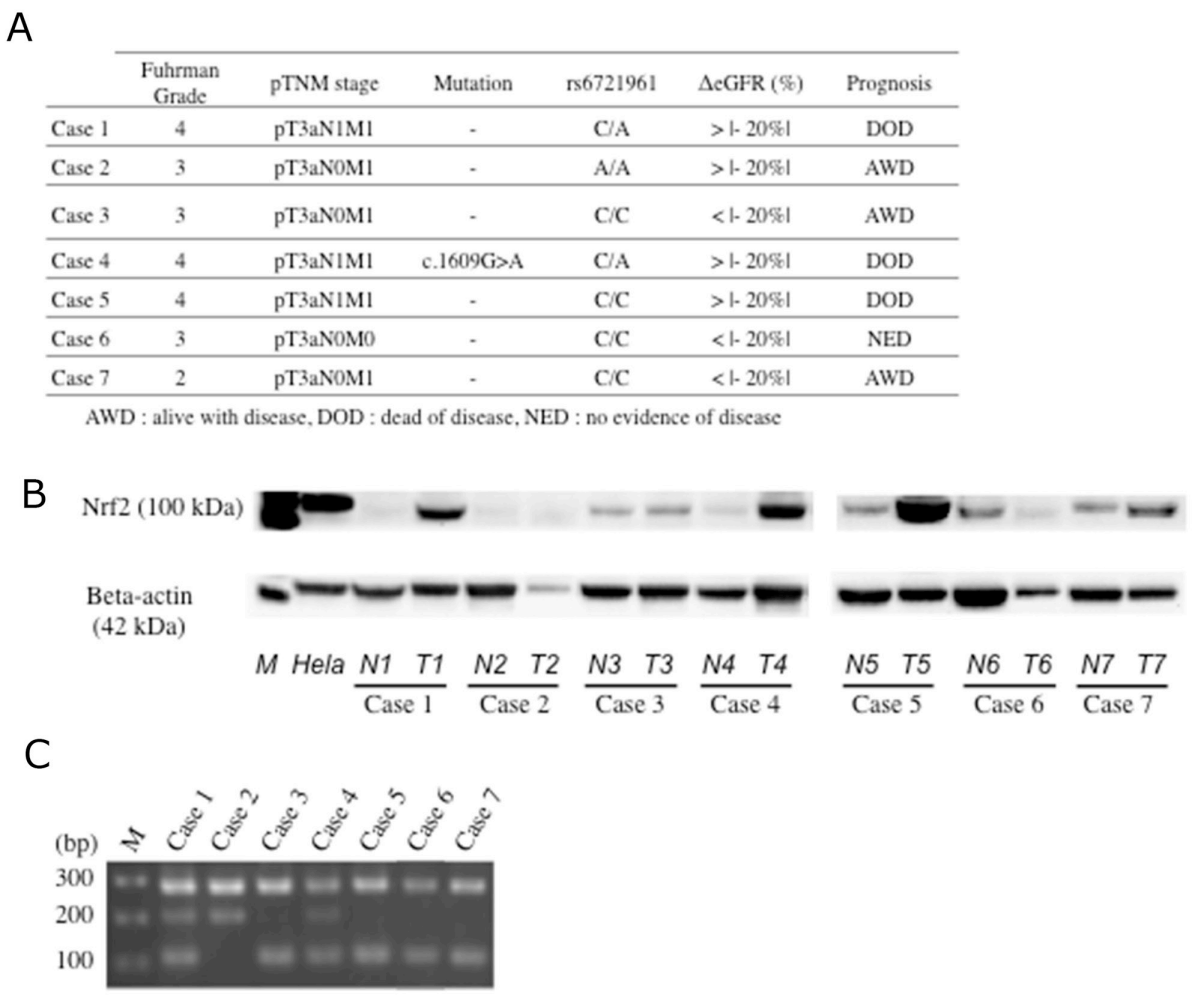
Expression of Nrf2 and genotyping of rs6721961 SNP (**A)** Clinical and pathological characterization of 7 patients analyzed. **(B)** Nrf2 protein was highly expressed in the primary tumor tissues than in non-tumor tissues. N; non-tumor tissue. T; primary tumor tissue. Each number corresponds to a case number. **(C)** Gel showing the genotype for rs6721961 SNP of the *Nrf2* gene. C/C genotype (282, 113 bp, in case 3, 5, 6 and 7), C/A genotype (282, 205, 113 bp, in case 1 and 4), and A/A genotype (282, 205 bp, in case 2).

**Figure 5:**
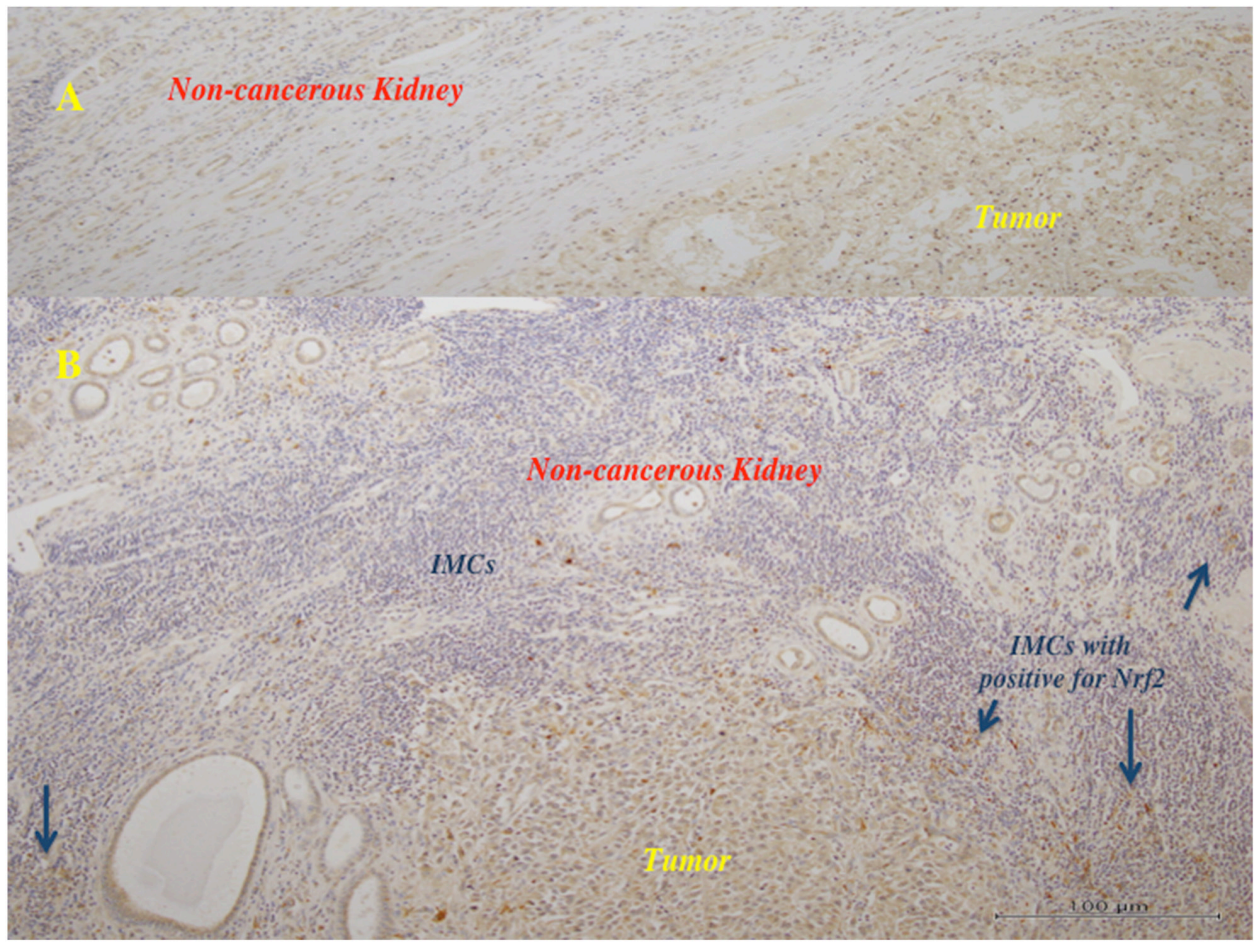
Immunohistochemistry in the primary tumor tissues for Nrf2 The tumor cells showed positive staining for anti-Nrf2 antibody. **(A)** (x100): In the tumors with lower histological grade (Fuhrman grade 1/2), many of the tumor cells showed weak to moderate reaction for anti-Nrf2 antibody, and the surrounding non-tumor tissues showed negative to very weak reaction. There were little infiltrating immune (mononuclear) cells (IMCs). **(B)** (x100): In the tumors with higher histological grade (Fuhrman grade 3), much of the tumor cells showed moderate to strong brown staining, and some IMCs showed strong reaction (arrows).

Nrf2 protein was detected in both tumor tissues and non-tumor tissues (Figures 4, 5), but its expression was significantly higher in tumor tissues (mean ± S.D. = 2.5 ± 1.1) compared with non-tumor tissues (expression arbitrarily set at 1.0) [15, 16]). Increased Nrf2 expression in the primary tumor was significantly associated with less differentiated histology (grade 1: 1.7 ± 0.9, grade 2: 2.1 ± 1.1, and grade 3: 3.1 ± 1.01, P=0.0046), local invasion (pT1/2: 1.7 ± 0.6 and pT3/4: 2.9 ± 1.2, P = 0.0035), microscopic vascular invasion (v0: 2.1 ± 1.1 and v1: 2.8 ± 1.2, P = 0.0422), and distant metastasis (M0: 1.8 ± 1.1 and M1: 3.8 ± 1.4, P = 0.0430).

Nrf2 expression was higher in the primary tumors of patients who had cardiovascular disease, hypertension, or diabetes than in the tumors of patients without these comorbidities (3.1 ± 0.6 vs. 2.2 ± 1.0, respectively, P = 0.0162).

On immunohistochemistry, many of the tumor cells in the RCCs with a lower histological grade (Fuhrman grade 1/2) were weakly to moderately positive for anti-Nrf2 antibody, while the surrounding non-tumor tissues with few infiltrating mononuclear cells (IMCs) showed a negative to very weak reaction (Figure 5A). In the RCCs with a higher histological grade (Fuhrman grade 3), many of the tumor cells showed moderate to strong positivity for anti-Nrf2 antibody, and some IMCs in the tumor and the surrounding non-cancerous kidney tissue were also strongly labeled (Figure 5B).

### Association of Nrf2 expression with changes eGFR or SUA

Nrf2 expression was higher in the primary tumors of patients with a decrease of eGFR by > 20% at 1 year after nephrectomy than in the tumors of patients with a decrease of eGFR by < 20% (3.1 ± 1.2 vs. 1.7 ± 0.8, respectively, P=0.0011). Similarly, tumor expression of Nrf2 was higher in patients with an increase of SUA by > 10% than in those with an increase of SUA by < 10% (2.9 ± 1.1 vs. 2.2 ± 1.1, respectively, P = 0.0355) (Table 2).

**Table 2:** Association of Nrf2 with change rate of eGFR and serum UA and pathologic characteristics

	|ΔeGFR (%)|^*^		p value	|ΔUA (%)|^*^		p value
	> |- 20%|	< |- 20%|		>10%	< 10%	
	mean ± S.D	mean ± S.D		mean ± S.D	mean ± S.D	
**Nrf2 in the primary tumor**	3.1 ± 1.2	1.7 ± 0.8	0.0011	2.9 ± 1.1	2.2 ± 1.1	0.0355
$$\text{Decreased ratio of eGFR}{\left(\text{eGFR}\right)}^{*}=\frac{\text{preoperative}-\text{postoperative serum level}}{\text{preoperative serum level}}\text{×}100\left(\%\right)$$							
$$\text{Increased ratio of UA}{\left(\text{UA}\right)}^{*}=\frac{\text{preoperative}-\text{postoperative serum level}}{\text{preoperative serum level}}\text{×}100\left(\%\right)$$							

	Nrf2 in the primary tumor		p value
	High	Low	
	mean ± S.D	mean ± S.D	
ΔeGFR (%)^*^	-30.8 ± 1.2	-20.4 ± 6.9	< 0.0001
ΔUA (%)^*^	24.2 ± 12.9	12.0 ± 8.2	0.0002
$$\text{Decreased ratio of eGFR}{\left(\text{eGFR}\right)}^{\text{*}}=\frac{\text{preoperative}-\text{postoperative serum level}}{\text{preoperative serum level}}\text{×}100\left(\text{\%}\right)$$							
$$\text{Increased ratio of UA}{\left(\text{UA}\right)}^{\text{*}}=\frac{\text{preoperative}-\text{postoperative serum level}}{\text{preoperative serum level}}\text{×}100\left(\text{\%}\right)$$							

### Prognostic impact of Nrf2

**Figure 6:**
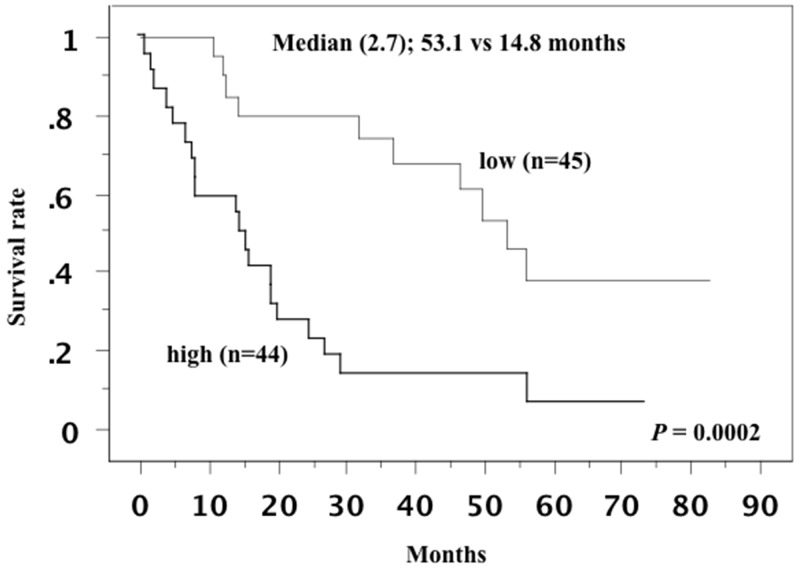
Overall survival curve in all patients This survival curve is based on the median value of Nrf2 in the primary tumor. The cases were divided into two groups at this levels: high and low expression. The patients with higher Nrf2 expression in the primary tumor showed shorter overall survival.

On western blotting, the median level of Nrf2 expression in the primary tumors was 2.7. The patients were divided into a high expression group (n=44) and a low expression group (n=45) at this cut-off value, as described previously [15, 16]. Kaplan–Meier plots for the patients with low or high Nrf2 expression showed that elevated Nrf2 expression in the primary tumor was associated with shorter overall survival (P = 0.0002, Figure 6). Univariate analysis of overall survival using the Cox proportional hazards model revealed that tumor grade, pT stage, microscopic vascular invasion, distant metastasis, and Nrf2 expression in the primary tumor were all significant variables (Table 3). Multivariate analysis confirmed that distant metastasis and Nrf2 expression were independent predictors of a worse prognosis.

**Table 3:** Cox regression analysis for various potential prognostic factors in overall survival

Variable	Unfavorable/favorable characteristics	No. of Patients	Univariate (U)			Multivariate (M)		
			Relative risk	95% confidential interval	P value	Relative risk	95% confidential interval	P value
Grade	3 / 2 / 1	51 / 29 / 9	2.376	1.279 - 4.412	0.0061	1.523	0.735 - 3.156	0.2580
pT	4, 3 / 2, 1	67 / 22	4.297	1.509 - 12.236	0.0063	1.368	0.432 - 4.331	0.5941
pV	1 / 0	78 / 11	2.557	0.890 - 7.347	0.0811	2.753	0.783 - 8.679	0.1144
M	1 / 0	64 / 25	4.224	1.275 - 13.997	0.0184	6.390	1.733 - 23.569	0.0053
Nrf2	high / low	44 / 45	4.671	2.173 - 10.038	< 0.0001	5.160	1.895 - 14.052	0.0013

### Molecular genetic analysis

Among 7 patients with locally advanced tumors and distant metastases who consented to analysis of Nrf2 mutations in germline and somatic DNA, one patient had a single nucleotide polymorphism (SNP) on exon 5 (position; 178,095,722 bp, genotype; c.1609G>A, Amino Acid; p.Glu537Lys) in the resected primary cancer, but not in peripheral blood leukocytes (Figure 4).

We also examined an SNP of the Nrf2 gene promoter region (rs6721961) in tumor samples from the 7 patients by using the real-time polymerase chain reaction with confronting two-pair primers (PCR-CTPP) (Figure 4). The genotype frequencies of rs6721961 were 57.1% for C/C, 28.6% for C/A, and 14.3% for A/A.

## DISCUSSION

The Nrf2/ARE signaling pathway is important for induction of antioxidant gene expression, and activation of Nrf2 plays a key role in counteracting oxidative stress. There is accumulating evidence that impaired activation of Nrf2 promotes oxidative stress associated with the onset of CKD [7], and there is also evidence that Nrf2 has a dual role in relation to malignancy and can either suppress carcinogenesis or support progression of cancer [12–14]. The present study showed that the risk of new-onset CKD was significantly higher in patients undergoing radical nephrectomy, particularly patients whose tumors showed higher Nrf2 expression, than in patients undergoing partial nephrectomy. Higher Nrf2 expression by the primary tumor was also associated with an unfavorable prognosis and was an independent prognostic determinant of shorter overall survival. These findings suggested that tumor overexpression of Nrf2 was associated with postoperative development of CKD and worse survival in patients with advanced RCC.

The important clinical issues are the implications of elevated Nrf2 expression in tumor tissues and how it influences normal renal tissue in the affected kidney or the contralateral kidney. In general, glomerular disease would be expected to progress simultaneously in both kidneys. If an RCC patient does not have glomerular and/or ureteral disease requiring medical treatment and presents with a relatively large renal tumor (>10 cm in diameter), particularly a tumor that has replaced most of the affected kidney, the contralateral normal kidney is almost totally responsible for renal function. In this setting, it is likely that resection of the affected kidney will not have much influence on the function of the preserved kidney. In contrast, nephrectomy will lead to a decrease of renal function in patients who have relatively small tumors (especially T1 tumors ≤ 4 cm in diameter), because the affected kidney contains a considerable amount of viable non-cancerous tissue. Considering both oncological and quality-of-life outcomes, it is therefore recommended that localized RCC (T1) should be managed by partial nephrectomy rather than radical nephrectomy, if this is technically feasible [17]. On the other hand, pre-existing occult nephropathy and/or other factors, including arteriosclerosis and endothelial injury, may also influence the function of the non-cancerous renal tissue in the affected kidney and the contralateral kidney. Thus, kidney function may decline before or after surgery in patients with pre-existing glomerular and/or ureteral disease requiring medical treatment. In the present study, eGFR decreased postoperatively in the patients with cardiovascular disease, hypertension, or diabetes, and Nrf2 expression was higher in the tumor tissues of those patients. Because activation of the Nrf2/ARE axis has a strong antioxidant effect, pharmacological activation of this axis has been suggested as a promising treatment for various diseases, including renal disease [8, 9]. In patients with such diseases, eGFR could decrease over time regardless of renal surgery. These patients have chronic inflammation, which might lead to a protective increase of Nrf2 expression, contrary to the suggestion that elevation of Nrf2 has a deleterious effect on renal function. On the other hand, we found that elevated tumor expression of Nrf2 was associated with a larger postoperative decrease of eGFR and a larger postoperative increase of SUA compared to the changes in patients with lower tumor expression of Nrf2. These observations support the notion that Nrf2 has a role in oxidative stress and inflammation associated with CKD.

From the results of western blotting and immunohistochemistry, it seems likely that increased Nrf2 expression in tumor tissues reflects its synthesis by both tumor cells and IMCs that were positive for Nrf2 antibody. It is well known that RCC is an immunogenic tumor. In the present study, we found that IMCs were prominent in the tumors with a higher histological grade (Fuhrman grade 3), but not in the lower grade tumors (Fuhrman grade 1/2). Also, Nrf2 expression was increased in the tumor cells and IMCs of Fuhrman grade 3 tumors. Furthermore, eGFR showed a greater decrease and survival was worse after radical nephrectomy in patients with tumors showing elevated Nrf2 expression. In contrast, patients who underwent partial nephrectomy for small RCCs ≤ 4 cm in diameter with a lower histological grade (Fuhrman grade 1/2) had few IMCs, lower expression of Nrf2 in the tumor and non-cancerous kidney tissues, and a smaller postoperative decrease of eGFR without progression to CKD. Among the patients undergoing radical nephrectomy, Nrf2 expression was higher in the non-cancerous kidney tissues of those whose tumors had a higher histological grade (Fuhrman grade 3) and numerous IMCs than in the non-cancerous tissues of those with lower grade tumors (Fuhrman grade 1/2) and few IMCs. This suggests that the extent of IMC infiltration into the tumor and surrounding non-cancerous kidney tissues is related to the biological aggressiveness of RCC, but the effect of up-regulated Nrf2 expression in IMCs is unclear. Further investigation is required to determine whether up-regulation of Nrf2 in tumor cells and IMCs influences the progression of RCC cooperatively or independently.

It is important to achieve better understanding of the mechanisms underlying overexpression of Nrf2 in RCC. While activation of Nrf2 may occur secondary to dysregulation of tumor suppressor genes and oncogenic pathways in cancer cells [13, 14], the precise association of Nrf2 with progression of this cancer is unclear. Mitsuishi et al. recently reported that increased Nrf2 expression redirects glucose and glutamine towards anabolic pathways, especially in the presence of sustained activation of phosphatidylinositol 3‘kinase (PI3K) - serine/threonine kinase Akt signaling. They also stated that activation of the PI3K-Akt pathway augments nuclear accumulation of Nrf2 and supports cell proliferation, as well as enhancing cytoprotection, indicating that Nrf2 activation promotes metabolic reprogramming triggered by proliferative signals [11]. Human cancers show activation of the PI3K, Akt, and mammalian target of rapamycin (mTOR) pathway. In particular, mTOR complex 2 (mTORC2) – phosphorylated Akt (Serine-473) signaling promotes utilization of glucose to provide energy for cell proliferation and survival [18], and has a very important role in RCC [19]. We recently reported that increased expression of phosphorylated Akt (Serine-473) in the primary tumor was correlated with the invasive and metastatic potential of RCC, as well as with a worse response of metastatic disease to vascular endothelial growth factor targeting therapy and an unfavorable prognosis [15, 16]. Since oxidative phosphorylation is impaired in RCC [20], co-activation of the Nrf2 pathway and the PI3K/Akt/mTOR pathway might cooperatively cause a shift of metabolism to aerobic glycolysis that enhances tumor cell growth. Thus, cross-talk between Nrf2 and the PI3K/Akt/mTOR pathway should be investigated in the future.

Somatic mutations of Nrf2 have been reported in many human cancers, and tumorigenic mutations typically result in activation of Nrf2 targets, emphasizing the importance of this molecule [13]. In the present study, we were able to examine tissue samples from 7 patients with locally advanced tumors and distant metastases, and we found an SNP at exon 5 of the Nrf2 gene in one patient. This SNP might have promoted Nrf2 expression, as indicated by western blotting, but it was a missense mutation and it seems difficult for it to influence Nrf2 expression. On the other hand, there reported that certain SNP (rs6721961) at promoter region of Nrf2 gene (Nrf2 regulatory SNP (rSNP)-617) is associated with Nrf2 expression. A recent study demonstrated that minor A/A homozygotes for rs6721961 exhibited lower Nrf2 gene expression and an increased risk of lung cancer [21]. Compared to the patients with C/C homozygous tumors, three patients with other tumor genotypes (two tumors with the C/A genotype and one with the A/A genotype) showed a larger decrease of eGFR and worse survival after nephrectomy. However, the present findings need to be confirmed in a larger cohort. We should also investigate the role of Nrf2 expression by IMCs in the tumor microenvironment to determine whether increased Nrf2 expression by these cells suppresses or promotes cancer progression. To elucidate the role of Nrf2 in cytoprotection and/or cancer progression, we need study its effects in both tumor cells and IMCs.

In conclusion, our findings suggested that activation of Nrf2 might play a role in postoperative new-onset CKD and the development/progression of RCC. However, this study had several limitations, including its retrospective design, a relatively small sample size, and a follow-up period too short to allow definite conclusions. Despite these limitations, our findings suggest that the antioxidant effect of Nrf2 in cancer cells could contribute to a worse outcome, so Nrf2 might be a two-edged sword. Therefore, it is important to elucidate the mechanisms regulating Nrf2 expression.

## MATERIALS AND METHODS

### Patients

This study retrospectively investigated 89 patients (68 men and 21 women) who did not have pre-existing chronic kidney disease (eGFR < 60 mL/min/1.73 m^2^) and underwent nephrectomy for clear cell RCC (ccRCC) between 2010 and 2015 at our hospital. The glomerular filtration rate was estimated by calculating eGFR using the abbreviated Modification of Diet in Renal Disease (MDRD) study equation [22]. All patients routinely underwent computed tomography (CT) and/or magnetic resonance imaging (MRI) for staging before surgery. The postoperative follow-up period ranged from 3 to 91 months (median: 19 months). Surgery was performed before patients received any other therapy. In each patient, three tumor tissue specimens and various non-neoplastic tissue specimens were harvested during surgery and were frozen at -80°C as soon as possible [15]. The clinical stage was determined according to the TNM classification [23]. This study was conducted in accordance with the Declaration of Helsinki and was approved by the ethical review board of Dokkyo Medical University Hospital. Before undergoing nephrectomy, each patient signed a consent form that was approved by our institutional Committee on Human Rights in Research.

### Western blotting and immunohistochemistry

Samples of tumor tissue and normal tissue were carefully dissected free of stromal tissue. Western blotting was performed with an anti-Nrf2 monoclonal antibody (Abcam, # ab-62352, Cambridge, UK), as described previously [15]. After protein bands were visualized by chemiluminescence, the membranes were scanned for densitometry with a PDI imaging scanner (Agfa Japan, Tokyo) and data were analyzed by using NIH Image software (ImageJ for Mac OS, version 1.50). Expression of Nrf2 was calculated relative to that of beta-actin in the tumor tissue specimens and corresponding non-neoplastic tissue specimens. Densitometric analysis was employed for semiquantitation of Nrf2 expression, with the relative amount of Nrf2 protein in each tumor tissue specimen being expressed as a ratio of the optical density for the tumor specimen to that for the corresponding non-neoplastic specimen (which was set at 1.0) [16].

To confirm the results of western blotting, representative tumor specimens from 7 patients were subjected to immunohistochemical analysis with the same antibodies utilized for western blotting [24].

### Next-generation sequencing

We only investigated Nrf2 mutations in 7 patients who gave written consent to analysis of germline and somatic DNA by signing a form that was approved by our institutional Committee on Human Rights in Research. Clinical samples were obtained, and sequencing and data analysis were conducted, as described previously [24]. In brief, germline DNA was extracted from leukocytes according to the standard protocols. Frozen tumor samples were ground to a powder in liquid nitrogen and 30–50 mg of the powder was used for DNA extraction with an AllPrep kit (Qiagen). DNA was quantified and its purity was assessed with a NanoDrop ND-1000 spectrophotometer (Labtech). We investigated Nrf2 mutations in the 7 patients by sequencing the coding exons and intron flanking regions in both peripheral blood leukocytes and tumor samples. For targeted next-generation sequencing analysis, customized primers were designed for the Nrf2 region by using Ampliseq Designer (Life Technologies). Construction of a library and sequencing were carried out using an Ion AmpliSeq Library Kit 2.0, Ion PGM IC 200 kit, and Ion PGM (Life Technologies) according to the manufacturer’s instructions. After each sequencing reaction, the raw data were analyzed with Torrent Suite version 4.2.1.

### Real-time PCR

Genotyping of an SNP in the promoter region of the *Nrf2* gene (rs6721961) was performed by PCR-CTPP, as described previously [25], using the following primers: forward primer 1: CCCTGATTTGGAGGTGCAGAACC, forward primer 2: GGGGAGATGTGGACAGCG, reverse primer 1: GCGAACACGAGCTGCCGGA, reverse primer 2: CTCCGTTTGCCTTTGACGAC. PCR was done with initial denaturation at 95°C for 10 min, followed by 30 cycles at 95°C for 1 min, 58°C for 1 min, and 72°C for 1 min, and final elongation at 72°C for 5 min. PCR products were resolved by electrophoresis on 2% agarose gel. Genotyping was performed for the CC genotype (282 and 113 bp), CA genotype (282, 205, and 113 bp), and AA genotype (282 and 205 bp).

### Statistical analysis

Western blotting data were analyzed by the Mann–Whitney U test for comparisons between two groups, while the Kruskal-Wallis test was employed to compare data among three groups. Associations between tumor pathological characteristics and the postoperative changes of eGFR and SUA were analyzed by Pearson’s χ^2^ test for contingency tables, while Spearman’s rank correlation coefficient analysis was performed to assess correlations between variables of interest. The Kaplan–Meier method was used to estimate survival and the significance of differences in survival was determined by the log-rank test. The impact of Nrf2 expression, tumor grade, T stage, vascular invasion, and metastasis on survival was assessed by both univariate and multivariate Cox proportional hazards analysis. All analyses were performed with commercially available software, and P < 0.05 was considered to indicate significance.
